# The effect of gender, age, and body mass index on the medial and lateral posterior tibial slopes: a magnetic resonance imaging study

**DOI:** 10.1186/s43019-021-00095-2

**Published:** 2021-04-08

**Authors:** Wazzan S. Aljuhani, Salman S. Qasim, Abdullah Alrasheed, Jumanah Altwalah, Mohammed J. Alsalman

**Affiliations:** 1 Department of Surgery, Ministry of the National Guard - Health Affairs, Riyadh, Saudi Arabia; 2 King Abdullah International Medical Research Center, Riyadh, Saudi Arabia; 3 King Saud bin Abdulaziz University for Health Sciences, Riyadh, Saudi Arabia; 4 Department of Radiology, Ministry of the National Guard - Health Affairs, Riyadh, Saudi Arabia

**Keywords:** Knee, MRI, Posterior tibial slope, Gender, Age, BMI

## Abstract

**Background:**

The posterior tibial slope (PTS) is crucial in knee joint stability and in maintaining the natural movement of the knee. An increase in the PTS is associated with various knee pathologic conditions, such as anterior cruciate ligament (ACL) injury and anterior tibial translation (ATT). In the present study, we aimed to establish native medial and lateral PTS values for adult Saudis and to identify any association between PTS and gender, age, and body mass index (BMI).

**Materials and methods:**

A total of 285 consecutive, normal, magnetic resonance imaging (MRI) studies of the knee were included in the study. The PTS was measured using the proximal anatomical axis of the tibia. The Kruskal-Wallis test was used to compare the medial and lateral PTS angles between age groups. The difference between the medial and lateral posterior tibial slopes was assessed using the Wilcoxon signed-rank test. The Mann-Whitney *U* test was performed to compare the medial and lateral PTS angles between men and women. Age, gender, and BMI were analyzed by multivariate linear regression to determine whether they positively predict the medial and lateral PTS angles.

**Results:**

The mean physiological medial PTS was 5.86 ± 3.0° and 6.61 ± 3.32°, and the lateral PTS was 4.41 ± 3.35° and 4.63 ± 2.85° in men and women, respectively. This difference showed no statistically significant gender dimorphism (*p* > 0.05). The medial PTS was significantly larger than the lateral PTS (*p* < 0.0001). There was no statistically significant difference in the medial and lateral PTS angles between age groups (*p* > 0.05). Higher BMI was significantly associated with a steeper medial PTS (*p* = 0.001).

**Conclusions:**

This study provided native values for medial and lateral PTS angles in Saudis, which can assist surgeons in maintaining normal knee PTS during surgery. The PTS was not influenced by age. The medial PTS was significantly larger than the lateral PTS in men and women. The PTS showed no significant gender dimorphism. BMI was significantly associated with the medial PTS.

## Background

The angle formed between the tibial longitudinal axis and the posterior inclination of the proximal surface of the tibia is defined as the posterior tibial slope (PTS) [1]. PTS is crucial in knee joint stability and in maintaining the natural movement of the knee, particularly knee flexion [2, 3]. An alteration in the PTS angle changes the mechanical axis of the natural knee, which is essential in maintaining normal knee kinematics [3, 4]. There is an increased incidence of anterior cruciate ligament (ACL) rupture with an increased PTS [5]. A study conducted by Matthias et al. [6] found that when the PTS angle increased by 1°, the chances of sustaining an ACL injury increased by 12%. Moreover, there is a significant increase in the possibility of sustaining an anterior tibial translation (ATT) with an increased PTS in both the active and passive movements of the knee [7].

Multiple PTS measurement methods have been reported, and they differ based on the joint reference axes used [8]. The tibial proximal anatomic axis (TPAA) and posterior tibial cortex (PTC), however, were shown to be more reliable than other reference axes, and the morphometric variables did not affect the values obtained using these two methods [8]. Differences in the medial and lateral PTS angles have been recorded in a cadaveric study [9]. It may be inappropriate to consider the PTS angle as a single value rather than two separate readings during arthroplasty. The PTS of the lateral and medial tibial compartments should be maintained during a high tibial osteotomy [10] or the implantation of a knee prosthesis [11].

Ethnicity has been reported to affect the PTS value in many studies of different ethnic groups [12–14], however, the findings of most of these studies were based on a single PTS value, measured on radiographs without two separate measurements for the medial and lateral PTS angles. Lateral-projection knee radiographs are routinely used to assess the PTS angle, but it is difficult to distinguish between the medial and lateral tibial plateaus using only conventional 2D radiographs [15], and many studies have highlighted this obstacle in their study limitations [12, 13, 15]. In addition, tibial rotation and inappropriately superimposed femoral condyles may result in imprecise measurement of the PTS on radiographs [16]. Due to limited resources to provide medial and lateral PTS values in the normal knee, the main goal of this study was to determine the PTS angle of the medial and lateral compartments of the tibial plateau in the normal knee in a population of adult Saudis, using magnetic resonance imaging (MRI), and to identify any association between the angle and gender, age, and body mass index (BMI).

## Materials and methods

This retrospective cohort study was conducted at King Abdulaziz Medical City (KAMC) in Riyadh, Saudi Arabia. A total of 6159 MRI studies of the knee in adult Saudis evaluated at KAMC between 1 March 2016 and 1 March 1 2020 were reviewed using the Centricity Enterprise (GE Healthcare Pvt. Ltd., Piscataway, NJ, USA) picture archiving and communication system (PACS). Only 285 consecutive knee MRI examinations out of the 6159 met the inclusion criteria and were included in the study. All the indications for the knee MRI, including traumatic and non-traumatic indications, were considered, and the magnetic resonance (MR) images were included if they met the inclusion criteria for our study. The inclusion criteria were male and female Saudi nationals, at least 18 years of age, who had undergone knee MRI with normal, non-pathological radiological findings confirmed by a radiologist. The exclusion criteria were the presence of fractures, meniscal injury, ligamentous injury, significant joint effusion, osteoarthritis changes, tumors, or previous knee procedures or implants. Knee MR images that showed any of the aforementioned conditions were excluded due to their potential association with the PTS.

**Fig. 1:**
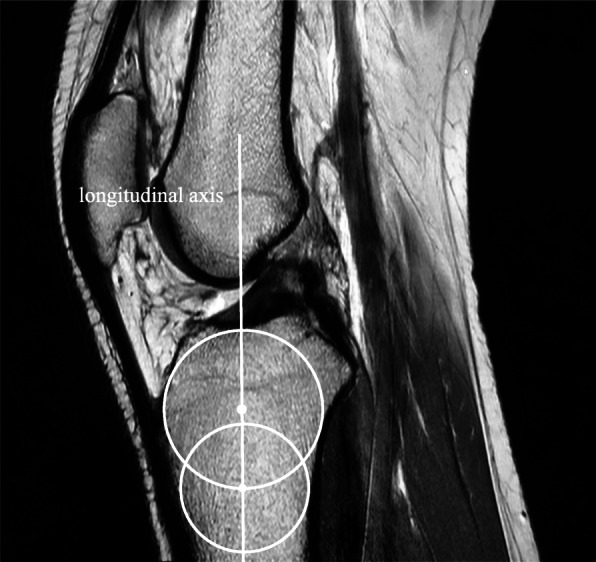
Central sagittal image showing the longitudinal axis of the tibia with two incorporated circles placed in the center of the tibial head

**Fig. 2:**
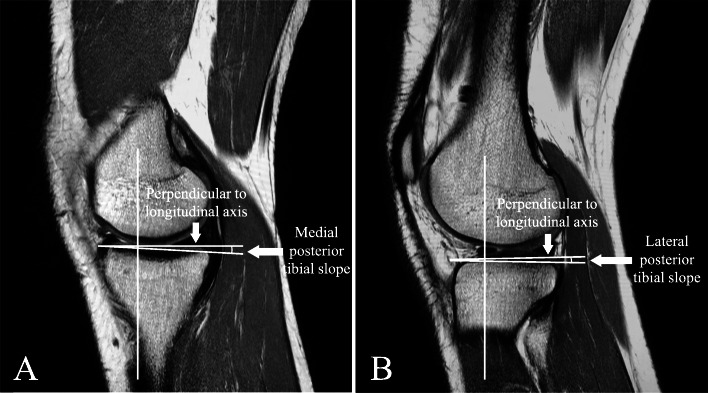
**a** Medial posterior tibial slope at the central sagittal plane of the medial tibial plateau. **b** Lateral posterior tibial slope at the central sagittal plane of the lateral tibial plateau

The BMI of each patient was obtained from the hospital’s electronic medical record database through the BESTCare system. According to the World Health Organization (WHO) general population classification, BMI was classified into four groups: < 18.5 kg/m^2^ - underweight, 18.5–24.9 kg/m^2^ - normal, 25.0–29.9 kg/m^2^ - overweight, and ≥ 30 kg/m^2^ - obesity. All knee MRI was obtained using the same standardized procedure of a proton-density, sagittal-plane sequence with a slice thickness 3 mm, echo time (TE) 30 ms, and repetition time (TR) 3700 ms. The researchers used the method described by Hudek et al. [17] for conventional MRI to measure the medial and lateral posterior tibial slopes, which consists of three steps. First, a central sagittal image was chosen; the central image was determined if it showed the tibial attachment of the posterior cruciate ligament (PCL), the intercondylar eminence, and the anterior and posterior tibial cortices appearing in a concave shape. Second, cranial and caudal circles were drawn at the tibial head. The advanced workstation (Advantage Workstation version 4.4, GE Healthcare, Milwaukee, WI, USA) was used to apply the circles. The application allows the user to draw all forms of shapes in every position and keep them in a fixed position in every slice of the image series. The cranial circle had to be in contact with the cranial tibial cortex, and the caudal circle had to have contact with the anterior and posterior cortical borders. The center of the caudal circle was at the circumference of the cranial circle. The final step consisted of drawing a vertical line through the center of both circles, which represented the longitudinal axis (Fig. 1). The sagittal images were correlated with the axial and coronal images, which guided the evaluators to determine the exact center of the lateral and medial tibial plateaus. Subsequently, the image series was sent to PACS. Using PACS tools, the slopes of both medial and lateral posterior tibial plateaus were calculated. This was done by drawing a line perpendicular to the longitudinal axis and a line tangential to the tibial plateau; the angle between the two lines represented the medial and lateral posterior tibial slopes (Fig. 2a and b). Proton density (PD) sequences were used to differentiate between the articular cartilage and the bone cortex. The cartilage maintains a bright signal, while the bone cortex exhibits a low signal. PD sequences were utilized to avoid mismeasuring the cartilage instead of the bone cortex.

Serial numbers were used for data entry instead of medical record numbers, to ensure patient anonymity. The data were entered and recorded on a secure Microoft Excel spreadsheet (Microsoft Corporation, Redmond, WA, USA) and were only accessed and utilized by the researchers. The participant’s informed consent was not required as the study was retrospective and patient data were anonymized. Ethical approval for the present study was obtained from the Institutional Review Board of King Abdullah International Medical Research Center, Ministry of National Guard-Health Affairs, Riyadh, Kingdom of Saudi Arabia (approval number RC20/457/R).

Two observers independently measured the medial and lateral posterior tibial slopes of the participants. An online tool (https://www.randomizer.org/) was used to randomly select 40 knee MRI studies for the assessment of intraobserver and interobserver reliability. The medial and lateral PTS angles of the 40 randomly selected knee MRI studies were measured by the two observers twice, 3 weeks apart. The intraclass correlation was used to calculate the intra and interobserver reliability. The intraclass correlation coefficient (ICC) for both intraobserver and interobserver reliability was excellent (> 0.8) for measurement of the medial and lateral PTS angles, representing strong agreement between measurements taken by the same observer and measurements compared between the two observers. ICCs for the medial and lateral PTS are shown in Tables 1 and 2, respectively.

**Table 1:** The intraobserver and interobserver reliability for measurement of the medial posterior tibial slope

	Observer 1	Observer 2	Interobserver reliability ICC
**First evaluation**			
Mean ± SD (°)	6.51 ± 3.20	6.51 ± 3.03	0.97510
**Second evaluation**			
Mean ± SD (°)	6.60 ± 3.33	6.50 ± 3.02	0.95907
**Intraobserver reliability ICC**	0.99145	0.97104	

*ICC* intraclass coefficient, *SD* standard deviation

**Table 2:** The intraobserver and interobserver reliability for measurement of the lateral posterior tibial slope

	Observer 1	Observer 2	Interobserver reliability ICC
**First evaluation**			
Mean ± SD (°)	5.02 ± 3.02	5.32 ± 2.73	0.87089
**Second evaluation**			
Mean ± SD (°)	5.53 ± 3.28	5.30 ± 2.89	0.87835
**Intraobserver reliability ICC**	0.91748	0.92564	

*ICC* intraclass coefficient, *SD* standard deviation

Although the ICC for interobserver reliability was excellent for measurement of both the medial and lateral PTS angles, there was larger variation in the measurement of the lateral PTS, as shown in Table 2. This can be attributed to the measurement technique used in the present study. For measuring the lateral PTS, the tangent was drawn on the uppermost even part of the tibial plateau between the superior-anterior and superior-posterior cortices, this measurement was more subjective and thus was more prone to variability between the two evaluators. In contrast, the tangent in the medial PTS was drawn connecting the uppermost anterior and posterior cortical edges of the proximal tibia, which were relatively more obvious the measurement was less subjective, thus was less prone to variability between the two evaluators.

Data in the present study were analyzed using the Statistical Analysis System, SAS (version 9.4). Continuous data are presented as mean and standard deviation (SD) and categorical data as frequency and percentages. The Kruskal-Wallis test was used to compare the medial and lateral posterior tibial slopes between age groups. The difference between the medial and lateral posterior tibial slopes was assessed using the Wilcoxon signed-rank test. The medial and lateral posterior tibial slopes in men and women were compared using the Mann-Whitney *U* test. Age, gender, and BMI were analyzed by multivariate linear regression to determine if they positively predict the medial and lateral PTS angles. All statistical tests were considered significant at *p* < 0.05.

## Results

Of the study sample, 187 (65.61%) were male and 98 (34.39%) were female. Participants were categorized by age as 18–24 years, 25–31 years, and 32–59 years, ensuring that the number of participants in each age group was as equal as possible to the other groups. A total of 10 participants were underweight, 88 (32.84%) were normal weight, 81 (30.22%) were overweight, and 89 (33.21%) were obese. The demographic information for the sample is displayed in Table 3. The mean physiological medial PTS was 5.86 ± 3.0° and 6.61 ± 3.32° and the lateral PTS was 4.41 ± 3.35° and 4.63 ± 2.85° in men and women, respectively (Table 4). The Kruskal-Wallis test showed no statistically significant difference between age groups in the medial and lateral PTS angles (Table 5). The Wilcoxon signed-rank test indicated a statistically significant difference between the medial and lateral PTS angles, with the medial PTS significantly greater than the lateral PTS (*p* < 0.0001) (Table 6). The Mann-Whitney *U* test demonstrated no statistically significant gender dimorphism on comparison of the medial and lateral PTS angles in men and women (Table 7). Multivariate linear regression analysis indicated no association between age, gender, or BMI, and the lateral PTS. However, higher BMI was significantly associated with a steeper medial PTS (*p* = 0.001) (Table 8).

**Table 3:** Participants’ demographics

Gender	Male	Female	Overall
N (%)	187 (65.61)	98 (34.39)	285 (100)
**Age category** N (%)			
18-24 years	53 (28.34)	26 (26.53)	79 (27.72)
25-31 years	70 (37.43)	33 (33.67)	103 (36.14)
32-59 years	64 (34.22)	39 (39.80)	103 (36.14)
**BMI** N (%)			
Underweight	6 (3.51)	4 (4.12)	10 (3.73)
Normal	57 (33.33)	31 (31.96)	88 (32.84)
Overweight	52 (30.41)	29 (29.90)	81 (30.22)
Obese	56 (32.75)	33 (34.02)	89 (33.21)

*BMI* body mass index

**Table 4:** Mean values and standard deviations of medial and lateral PTS angles according to gender

PTS angle (°)	Male (***n*** = 187)	Female (***n*** = 98)	Overall (***n*** = 285)
Medial PTS	5.86 ± 3.03	6.61 ± 3.32	6.12 ± 3.14
Lateral PTS	4.41 ± 3.35	4.63 ± 2.85	4.49 ± 3.19

*PTS* posterior tibial slope

**Table 5:** Comparison between medial and lateral PTS angles across age groups

	Age category			
	18–24 years	25–31 years	32–59 years	*P* value
**Medial PTS (°)**				
**Male (*n*** **= 187)**	5.9 ± 2.98	5.64 ± 3.27	6.07 ± 2.8	0.6549
**Female (*n*** **= 98)**	7 ± 4.14	6.29 ± 2.76	6.63 ± 3.19	0.8203
**Lateral PTS (°)**				
**Male (*n*** **= 187)**	5.04 ± 4.84	4.03 ± 2.5	4.31 ± 2.56	0.6800
**Female (*n*** **= 98)**	4.24 ± 2.39	4.57 ± 2.83	4.95 ± 3.16	0.7682

*PTS* posterior tibial slope

**Table 6:** The difference between the medial and lateral posterior tibial slope angles

	Medial PTS (°)	Lateral PTS (°)	***P*** value
**Male (*n*** **= 187)**	5.86 ± 3.03	4.41 ± 3.35	< 0.0001
**Female (*n*** **= 98)**	6.61 ± 3.32	4.63 ± 2.85	
**Overall (*n*** **= 285)**	6.12 ± 3.14	4.49 ± 3.19	

*PTS* posterior tibial slope

**Table 7:** Comparison between the medial and lateral PTS angles in men and women

	Male (***n*** = 187)	Female (***n*** = 98)	***P*** value
**Medial PTS (°)**	5.86 ± 3.03	6.61 ± 3.32	0.123
**Lateral PTS (°)**	4.41 ± 3.35	4.63 ± 2.85	0.389

*PTS* posterior tibial slope

**Table 8:** The effect of gender, age, and BMI on the lateral and medial PTS angles (multivariate regression)

	Predictor	Coefficients			Regression	
		Beta	***t***	***P*** value	***F*** score	***P*** value
**Lateral PTS**	(Constant)	–	2.622	0.009	1.143	0.332
	Gender	0.022	0.359	0.720		
	Age	- 0.028	- 0.457	0.648		
	BMI	0.110	1.781	0.076		
**Medial PTS**	(Constant)	–	2.784	0.006	5.421	0.001
	Gender	0.009	1.650	0.100		
	Age	- 0.073	- 1.213	0.226		
	BMI	0.211	3.517	0.001		

*PTS* posterior tibial slope, *BMI* body mass index

## Discussion

Normal variation of the PTS is important for the maintenance of knee kinematics and stability [3, 4, 6]. An increase in the PTS is associated with various knee injuries, such as ACL sprain or rupture and ATT [5–7]. The PTS can vary significantly according to the reference point used for the measurement [8]. In the present study, the medial and lateral PTS were measured according to the method described by Hudek et al. [17], where the proximal anatomical axis of the tibia (MRI longitudinal axis) was used as a reference point. Several techniques have been introduced for the measurement of the PTS on conventional knee radiographs. However, distinguishing between the medial and lateral PTS angles on conventional radiographs is difficult and may lead to imprecise measurements [17]. Knee MRI is among the most common imaging studies ordered for various clinical indications such as pain and trauma, as they provide comprehensive images of structures within the knee joint. On the other hand, computed tomography (CT) provides less detailed images and is usually used for preoperaive and postoperative assessment and for specific indications that would most likely reveal knee abnormalies. Furthermore, compared to CT, MRI shows soft tissue images such as injured ligaments more clearly [18], which serves the purpose of this study in assessing medial and lateral PTS angles in the normal knee.

In addition to the method used for the measurement of the PTS, ethnicity largely determines what is considered normal for the PTS. Several studies based on various modalities have reported differing normal values for the PTS according to ethnicity [19, 20]. The findings of the present study demonstrated a mean physiological medial PTS of 5.86 ± 3.0° and 6.61 ± 3.32°, and lateral PTS of 4.41 ± 3.35° and 4.63 ± 2.85° in men and women, respectively. Table 9 shows the mean medial and lateral PTS angles in different ethnic groups, measured on MRI, using the proximal anatomical axis of the tibia as a reference axis.

**Table 9:** Comparison of the medial and lateral PTS angles among different ethnic groups using MRI

			Male		Female	
Authors	Population	Sample size	Medial PTS (°)	Lateral PTS (°)	Medial PTS (°)	Lateral PTS (°)
Cinotti et al*.* [21]	Italian	80	7.6 ± 3.3	7.5 ± 3.5	8.6 ± 2.6	8.0 ± 3.6
Han et al. [22]	South Korean	535	6.1 ± 1.7	6.9 ± 1.7	6.1 ± 1.8	6.8 ± 1.9
Hashemi et al*.* [15]	American	55	3.7 ± 3.1	5.4 ± 2.8	5.9 ± 3.0	7.0 ± 3.1
Hudek et al*.* [17]	Swiss	100	4.6 ± 2.4	5.0 ± 3.6	–	–
Ümeyir et al*.* [23]	Turkish	232	7.7 ± 1.2	7.3 ± 1.2	7.8 ± 1.4	7.6 ± 1.3
Current study	Saudi	285	5.86 ± 3.0	4.41 ± 3.35	6.61 ± 3.32	4.63 ± 2.85

*MRI* magnetic resonance imaging

It was hypothesized that the medial and lateral PTS would increase with advancing age due to potential degenerative changes that occur at the tibial plateau, rendering the elderly more susceptible to developing knee injuries associated with an increased PTS, such as ACL rupture and ATT. In the present study, however, age did not significantly influence the medial and lateral PTS in men or women. This finding is consistent with an MRI study in Iranians, which also found no significant correlation between age and the PTS [24]. Similarly, Hashemi et al. [15] and Han et al. [22] concluded in their MRI studies that age was not correlated with the PTS in skeletally mature participants, in agreement with other studies conducted in Italian and Turkish population subsets [21, 23]. In addition, in a study involving a large number of cadaveric specimens, authors found that age did not influence the medial or the lateral PTS [25]. This cadaveric study, however, was designed to include specimens from people under the age of 55 years, to ensure that the measurements of the tibial plateau were not influenced by osteoarthritic changes. The results of the MRI studies are in agreement with several radiographic studies that investigated the effect of age on the PTS in adults [7, 13], including a previous study conducted in the same ethnic subset (Saudis) [26].

It is well-documented that an increased PTS is associated with an increase in ACL strain and the development of an ACL injury [27–29]. A steeper PTS increases the likelihood of sustaining an ATT [7, 30]. Higher BMI was linked to an increased risk of ACL tear in the presence of a steeper lateral PTS [31], because a steeper PTS facilitates the tibia in translating anteriorly, which results in overtightening of the ACL, one of the main structures responsible for knee stability [2, 3]. It is possible that the extra load on the knee joint from obesity may increase the wear and tear process of the cartilage and bone surfaces of the knee, a primary weight-bearing joint, leading to alteration of the proximal tibia and subsequent modification of the PTS. If BMI is positively correlated with the PTS, then lowering it by losing weight may potentially reduce the risk of an ACL injury, and it could be used to identify individuals who are at risk of ACL injury. In the present study, we aimed to test the association between BMI and the PTS in the medial and lateral compartments of the tibia. The multivariate regression model showed that BMI was significantly associated with the medial PTS (*p* = 0.001), while the lateral PTS was not associated with age, gender, or BMI (Table 8). To our knowledge, there are no MRI studies assessing the relationship between BMI and the PTS. More studies are required to fully understand the relationship between BMI and the PTS of the medial and lateral tibial compartments.

A number of studies of the proximal tibia demonstrated a significant difference between the medial and lateral PTS angles [14, 15]. Hashemi et al. [15] reported a significant difference between the within-subject medial and lateral PTS angles in male and female participants in their MRI study, with the lateral PTS greater than the medial PTS in both genders. Khattak et al. [13] assessed the variation in the medial and lateral tibia plateau in healthy Pakistani volunteers on radiographs of the knee, and reported that the medial PTS was greater than the lateral PTS in the female, but not in the male volunteers. It is worth noting that they used the anterior tibial cortex (ATC) as a reference axis to conduct their measurements. In contrast, Hudek et al. [17] found no significant difference between the mean medial and lateral PTS angles in Swiss people, but their findings showed large variation in the PTS values. In the present study the medial PTS was significantly larger than the lateral PTS in male and female adult Saudis analyzed separately and as a combined group. This ethnic and gender-related variation may be attributed to genetic, sociocultural, and environmental factors, and to the technique and imaging modality used to measure the PTS [22]. For example, Saudis pray five times daily in an Islamic ritual that requires performing deep knee flexion. In addition, it is very common for Saudis to sit with their knees fully bent, given the setting in Saudi Arabia. In terms of economic factors, some individuals do not have access to conventional toilets and therefore tend to squat. These factors may have an influence on the PTS and other anthropometric measurements.

We found contradictory results in the literature when comparing medial and lateral PTS angles in men and women. In the present study, we found no significant gender dimorphism in Saudis. Specifically, the medial PTS was 5.86 ± 3.03° and 6.61 ± 3.32° and the lateral PTS was 4.41 ± 3.35° and 4.63 ± 2.85° in men and women, respectively. Cinotti et al. [20] and Han et al. [22] also reported no gender difference in medial and lateral PTS angles in Italian and South Korean participants. This is consistent with several radiographic studies conducted in different ethnic groups [2, 12, 26]. In contrast, Hashemi et al. [15] found that the medial and lateral PTS angles were greater in women compared to men, potentially rendering them more susceptible to developing an ACL injury and ATT [5–7]. In addition, Khattak el at [13]. found that the medial PTS was significantly greater in female compared with male participants in their radiographic study, with no gender variation in the lateral PTS.

Maintaining normal values of the medial and lateral PTS angles is important as they are vital in knee stability and kinematics [2–4]. A steeper PTS may cause the tibia to translate anteriorly, potentially leading to the development of an ACL injury [5–7]. The native values for the PTS in the present study are important for Saudis undergoing knee surgery, such as total knee arthroplasty (TKA) and high tibial osteotomy. The PTS affects knee joint stability, biomechanics, and PCL tension after TKA [27, 32], and a reduced PTS can lead to abnormal femoral rollback, polyethylene wear, and postoperative stiffness [32]. Moreover, TKA with excessive PTS results in an increased contact area, smaller contact pressure, and posterior femoral translation [33]. Therefore, the PTS greatly influences the clinical outcomes of TKA. Considering the PTS in ACL reconstruction surgery is also important, as a steeper PTS was found to be a risk factor for the failure of ACL reconstruction [34].

The findings of the present study may assist surgeons in identifying patients who are at increased risk for developing an ACL injury based on their medial and lateral PTS values, and in reconstructing a PTS that is close to the normal range to ensure optimal clinical outcomes. In addition to preoperative planning, knowledge of the native values of the PTS for Saudis may be useful in the restoration of normal knee kinematics during TKA [31]. Last, knee prosthesis companies may utilize the measurements for the manufacturing of knee prostheses for individuals of Saudi Arabian descent.

There were some limitations to the present study. First, participants’ height and weight were not recorded as separate values. Instead, they were presented as BMI. Height and weight may have an influence on the PTS when considered separately, irrespective of the overall effect of the BMI on the slope. Second, although measurements obtained from MRI are more accurate than those obtained from conventional radiographs, errors in identifying landmarks cannot be completely avoided. This is because of variations in image resolution and the fact that measurements are taken from the distal femur and proximal tibia instead of from the whole tibia. Last, the primary aim of the present study was to establish native values for the medial and lateral PTS in a Saudi population subset, and only normal knee MR images were assessed. PTS values may vary in abnormal knee joints as well as in the relationship between the PTS and other variables.

## Conclusions

This study provided native values for medial and lateral PTS angles in Saudis. The BMI was significantly associated with the medial PTS, potentially increasing the risk of developing an ACL injury and ATT. Age was not statistically significant in terms of the medial and lateral PTS. The medial PTS was significantly larger than the lateral PTS in men and women. Comparing the medial and lateral PTS angles between men and women, we found no significant gender dimorphism.

## Data Availability

The datasets used and/or analysed during the current study are available from the corresponding author on reasonable request.
